# Rabbit Hepatitis E Virus, Ukraine, 2024

**DOI:** 10.3201/eid3104.250074

**Published:** 2025-04

**Authors:** Sérgio Santos-Silva, Yevheniia Dudnyk, Oksana Shkromada, Maria S.J. Nascimento, Helena M.R. Gonçalves, Wim H.M. Van der Poel, António Rivero-Juarez, João R. Mesquita

**Affiliations:** School of Medicine and Biomedical Sciences, University of Porto, Porto, Portugal (S. Santos-Silva, J.R. Mesquita); Faculty of Veterinary Medicine, Sumy National Agrarian University, Sumy, Ukraine (Y. Dudnyk, O. Shkromada); Faculty of Pharmacy, University of Porto, Porto (M.S.J. Nascimento); Faculty of Sciences, University of Porto, Porto (H.M.R. Gonçalves); Wageningen University, Lelystad, the Netherlands (W.H.M. Van der Poel); Wageningen Bioveterinary Research, Lelystad (W.H.M. Van der Poel); Hospital Universitario Reina Sofia, Instituto Maimonides de Investigación Biomédica de Córdoba, Universidad de Córdoba, Cordoba, Spain (A. Rivero-Juarez); Center for Biomedical Research Network in Infectious Diseases, Health Institute Carlos III, Madrid, Spain (A. Rivero-Juarez); Center for the Study of Animal Science, Institute of Sciences, Technologies, and Agroenvironment, University of Porto, Porto (J.R. Mesquita); Associate Laboratory for Animal and Veterinary Science (AL4AnimalS), Lisbon, Portugal (J.R. Mesquita)

**Keywords:** hepatitis E virus, viruses, One Health, NGS, next-generation sequencing, rabbit HEV, zoonoses, Ukraine

## Abstract

We identified hepatitis E virus (HEV) 3ra, a potentially zoonotic subtype, in rabbits in Ukraine, which highlights their potential role as reservoirs. Screening of rabbit fecal samples identified HEV-3ra. Public health services in this country should improve HEV surveillance and expand HEV sampling.

Hepatitis E virus (HEV), family Hepeviridae, species *Paslahepevirus balayani*, is a major cause of acute enterically transmitted hepatitis worldwide, particularly in developing nations, where its public health impact is substantial (*1*). *P. balayani* has 8 genotypes (*2*). Zoonotic genotypes 3 and 4 are globally distributed and transmitted to humans through consumption of infected animal products or contact with infected animals, particularly pigs (*3*). Rabbits (*Oryctolagus cuniculus*) have been identified as reservoirs for HEV, specifically the potentially zoonotic HEV-3ra subtype linked to human infections, with cases reported during 2015–2016 (*4*). Despite widespread detection of HEV in Western and Central Europe, its prevalence and the role of animals in transmission remain underexplored in Eastern Europe, especially Ukraine. That knowledge gap is concerning because of the zoonotic risk from rabbit meat consumption or environmental exposure (*5*). The risk might be heightened in situations of armed conflict, where greater reliance on bushmeat could increase the chances of spillover events. We investigated the prevalence and molecular characterization of HEV in domestic rabbits from Ukraine.

In April 2024, we collected 108 fresh fecal samples from clinically healthy Californian domestic rabbits (*O. cuniculus*) born and raised on private family farms in the Sumy region, northeastern Ukraine. We suspended samples in 10% phosphate-buffered saline (pH 7.2) and centrifuged at 8,000 *× g* for 5 minutes. We extracted total nucleic acids from 200 µL of the supernatant by using the QIAamp Cador Pathogen Mini Kit (QIAGEN, https://www.qiagen.com) on a QIAcube automated system, according to the manufacturer’s protocol. For HEV RNA, we used a pangenotypic quantitative reverse transcription PCR (RT-PCR) targeting the conserved open reading frame (ORF) 3 region (*6*), then used a broad-spectrum nested RT-PCR targeting the ORF1 region (*7*). We used the World Health Organization PEI 6329/10 subgenotype 3a (GenBank accession no. AB630970) as a positive control. We performed real-time PCR on a CFX96 Real-Time PCR System (Bio-Rad Laboratories, https://www.bio-rad.com) by using the NZYSupreme One-Step RT-qPCR Probe Master Mix (2×) kit plus ROX dye (NZYTech, https://www.nzytech.com). We performed nested RT-PCR on a T100 thermocycler (Bio-Rad Laboratories) by using the Xpert One-Step RT-PCR Kit (GriSP, https://grisp.pt) in the first round and the Xpert Fast Hotstart Mastermix 2× with dye (GriSP) in the second round, according to the manufacturer’s instructions. We used Sanger sequencing to sequence the suspected positive amplicons in both directions. We aligned trimmed sequences by using Sequence Alignment Editor version 7.1.9 (BioEdit, https://bioedit.software.informer.com) and compared with nucleotide sequences from GenBank.

To further analyze positive samples, we used sequence-independent single-primer amplification with a previously described protocol (*8*), and the sequence-independent single-primer amplification samples underwent next-generation sequencing at Novogene (https://www.novogene.com) by using the NovaSeq 6000 platform (Illumina, https://www.illumina.com). We trimmed data by using BBduk version 38.86 (https://jgi.doe.gov/data-and-tools/software-tools/bbtools) to remove adaptor sequences and low-quality bases. We mapped the trimmed reads to the HEV reference genome by using BBMap version 38.86 (https://jgi.doe.gov/data-and-tools/software-tools/bbtools), processed the reads with Samtools version 1.18 (http://www.htslib.org) to generate binary alignment map files, and created a consensus FASTA sequence. We conducted phylogenetic analysis by using MEGA X software version 10.2 (https://www.megasoftware.net) and applied the maximum-likelihood method with 1,000 bootstrap replicates. We used the HEVnet tool (https://www.rivm.nl/en/hevnet) for further genotyping (*9*).

We detected HEV RNA in 1 (0.93%, 95% CI 0.02%–5.05%) rabbit stool sample from the 108 samples tested. We obtained 1 genome fragment from ORF2 and 2 fragments from ORF1 (Table). Sequence analysis confirmed the genome identity as HEV. The ORF2 fragment (GenBank accession no. PQ541216) showed identity with 2 human sequences from Switzerland: 92.04% identity with GenBank accession no. OX044324 and 91.72% with GenBank accession no. OV844765. One ORF1 fragment (GenBank accession no. PQ541214) showed identity with rabbit sequences from Australia: 91.42% with GenBank accession no. MW002522 and 90.99% with GenBank accession no. MZ676756. The other ORF1 fragment (GenBank accession no. PQ541215) displayed lower similarity to sequences from human samples from France (87.59% with GenBank accession no. MF444074) and rabbit samples from China (86.57% with GenBank accession no. KX227751).

**Table:** Details of HEV genome fragments obtained and analyzed in study of rabbit hepatitis E virus, Ukraine, 2024*

Sequence ID	Length, bp	Nucleotide start position	Nucleotide end position	GenBank accession no.
5	235	4263	4498	PQ541214
5	150	1765	1915	PQ541215
5	314	5836	6150	PQ541216

*Nucleotide start and end positions are relative to the NC_001434 reference sequence.

Phylogenetic analysis confirmed clustering of the obtained RNA-dependent RNA polymerase genome fragment to the rabbit-associated HEV-3ra (Figure). The HEV-3ra prevalence in rabbits reported in this study (0.93%) is lower than in another study from Europe, in which rabbit-associated HEV prevalence ranged from 7% to 23% in domestic and wild rabbits (*5*). However, the absence of HEV RNA has also been reported in other regions of Europe, such as in Portugal (*10*). Those differences could be attributed to regional variations in sampling and detection methods, environmental factors, and surveillance intensity.

**Figure:**
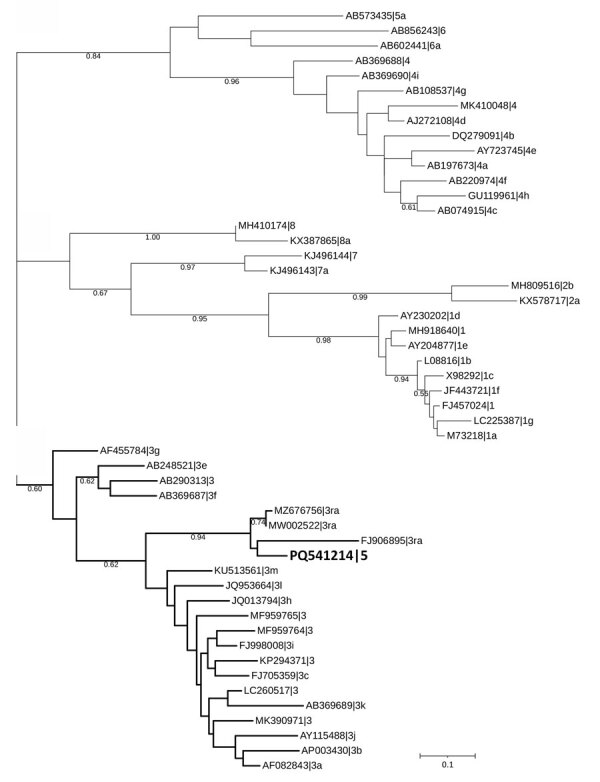
Phylogenetic tree of the RNA-dependent RNA polymerase sequence of rabbit hepatitis E virus (HEV), Ukraine, 2024. Bold indicates the HEV sequence identified in this study. The tree was inferred using MEGA X software (https://www.megasoftware.net) with the Kimura 2-parameter substitution model and visualized using the Interactive Tree of Life (https://itol.embl.de). The tree includes 51 HEV nucleotide sequences, with reference sequences retrieved from GenBank, displayed with their accession number, genotype, and subgenotype. The tree is structured into distinct clusters corresponding to different HEV genotypes and subgenotypes; the detected sequence groups within the HEV-3ra cluster. This placement indicates its close relationship to previously reported rabbit HEV sequences. Sequence analysis confirmed the genome identity as HEV. The ORF2 fragment (GenBank accession no. PQ541216) showed identity with 2 human sequences from Switzerland: 92.04% identity with GenBank accession no. OX044324 and 91.72% with GenBank accession no. OV844765. One ORF1 fragment (GenBank accession no. PQ541214) showed identity with rabbit sequences from Australia: 91.42% with GenBank accession no. MW002522 and 90.99% with GenBank accession no. MZ676756. Scale bar indicates nucleotide substitutions per site.

In summary, this study provides evidence for HEV circulation in Ukraine, specifically the potentially zoonotic HEV-3ra in a domestic rabbit, addressing a key gap in its epidemiology. The genetic similarity of the detected HEV-3ra strain to those found in humans and animals elsewhere highlights the subgenotype’s zoonotic potential and risk for cross-border transmission. Future research should expand sampling across species and regions using molecular and serologic studies to clarify transmission dynamics and public health risks. The findings in this study emphasize the need for robust surveillance in animals and humans to clarify HEV circulation.
